# Laboratory-Confirmed Hepatitis A, Hepatitis E, and Rotavirus Infections at a Tertiary Care Center in Eastern Rajasthan, India: A Prospective Observational Study

**DOI:** 10.7759/cureus.113348

**Published:** 2026-07-25

**Authors:** Asmita Kaur, Rohit Sachdev, Harshita Upadhyay

**Affiliations:** 1 Department of Microbiology, RVRS (Rajmata Vijaya Raje Scindia) Government Medical College, Bhilwara, IND

**Keywords:** acute gastroenteritis, acute hepatitis, hepatitis a, hepatitis e, rotavirus

## Abstract

Background

Hepatitis A virus (HAV) and hepatitis E virus (HEV) are important fecal-oral viral causes of acute hepatitis, whereas rotavirus is a major cause of acute gastroenteritis in India. Their occurrence varies by age, season, sanitation, and local exposure patterns. This prospective study assessed the laboratory positivity, temporal distribution, and clinico-epidemiological characteristics of HAV, HEV, and rotavirus infections among eligible patients tested at a tertiary care center in Eastern Rajasthan, India.

Methods

This prospective observational study was conducted at RVRS (Rajmata Vijaya Raje Scindia) Government Medical College, Bhilwara, Rajasthan, from January 2025 to March 2026. Eligible patients with clinically suspected acute viral hepatitis and/or acute diarrheal illness were enrolled consecutively. Demographic, socioenvironmental, clinical, and laboratory data were recorded using a structured case record form. Anti-HAV immunoglobulin M (IgM) and anti-HEV IgM antibodies were detected by enzyme-linked immunosorbent assay (ELISA), while rotavirus infection was confirmed by stool antigen testing.

Results

A total of 3,038 patients were tested for HEV, 2,574 for HAV, and 560 for rotavirus. Rotavirus showed the highest laboratory positivity, detected in 80/560 patients (14.3%; 95% confidence interval (CI): 11.5-17.4), followed by HAV in 350/2,574 patients (13.6%; 95% CI: 12.3-15.0) and HEV in 319/3,038 patients (10.5%; 95% CI: 9.4-11.7). Among the 749 laboratory-confirmed cases, the overall number of cases was highest during October-December 2025 (227/749, 30.3%). HEV cases peaked during July-September 2025 (113/319, 35.4%), HAV cases during January-March 2026 (123/350, 35.1%), and rotavirus cases during October-December 2025 (56/80, 70.0%). Mean age differed significantly across etiologies: HEV, 19.45 ± 10.31 years; HAV, 17.96 ± 13.49 years; and rotavirus, 4.21 ± 1.59 years (Kruskal-Wallis H = 99.64, p < 0.001). Males predominated among HAV (222/350, 63.4%) and rotavirus cases (50/80, 62.5%), whereas females predominated among HEV cases (199/319, 62.4%) (p < 0.001). Open defecation was reported more frequently among HAV (30.6%) and HEV (30.1%) cases than among rotavirus cases (12.5%) (p = 0.004). Nausea/vomiting, loss of appetite, jaundice, and dark urine were more common among HAV and HEV cases, whereas abdominal pain was most frequent among rotavirus cases (61.3%) (all p < 0.001).

Conclusions

HAV, HEV, and rotavirus contributed substantially to laboratory-confirmed enteric viral infections among eligible patients tested in Eastern Rajasthan. The three etiologies showed distinct seasonal, demographic, and clinical patterns. Continued prospective laboratory surveillance, timely diagnostic testing, safe water access, and improved sanitation and hygiene practices are needed to reduce the burden of these infections.

## Introduction

Viral hepatitis is a significant cause of morbidity in India and is considered a public health threat comparable to other major communicable diseases, including AIDS, malaria, and tuberculosis [1]. The World Health Organization estimates that approximately 1.4 million cases of hepatitis A occur globally each year, resulting in around 7,000 deaths [2]. In comparison, an estimated 20 million hepatitis E infections occur annually, leading to approximately 3.3 million symptomatic cases and 44,000 deaths [2]. Among enterically transmitted viral pathogens, hepatitis A virus (HAV) and hepatitis E virus (HEV) are epidemiologically important causes of acute viral hepatitis [3]. Both viruses may cause sporadic infections as well as epidemic outbreaks.

HAV is transmitted primarily through the fecal-oral route and is associated with poor sanitation and hygiene. HAV infection commonly occurs during childhood and often results in mild or anicteric hepatitis [4]. India is categorized as a hyperendemic country for HAV because exposure commonly occurs early in life, when infection is often subclinical. HEV is also an enterically transmitted pathogen that spreads mainly through the fecal-oral route [2]. It is an important cause of acute viral hepatitis and may result in epidemics, focal outbreaks, and sporadic infections [2]. During outbreaks, pregnant women have a higher likelihood of HEV infection and a greater risk of developing acute liver failure compared with non-pregnant women [5].

Diarrhea is an important cause of childhood morbidity and mortality globally and in India [6,7]. Rotavirus is a major cause of acute gastroenteritis in young children and can lead to severe dehydration if not managed promptly [8]. Rotavirus infection typically presents with acute watery diarrhea, vomiting, and fever, and symptoms usually resolve within approximately one week in uncomplicated cases. The burden of rotavirus disease varies according to geographic location, age, and other population characteristics [9,10].

Recent epidemiological studies from Rajasthan have consistently demonstrated that HAV, HEV, and rotavirus contribute substantially to the burden of laboratory-confirmed enterically transmitted viral infections. Soni et al. reported laboratory positivity of 12.7% for HAV and 10.9% for HEV among patients with suspected acute viral hepatitis, with most cases occurring during the monsoon and post-monsoon period and being associated with poor sanitation [2]. Similarly, Singh and Tiwari found that more than half of patients with suspected acute viral hepatitis had laboratory-confirmed HAV or HEV infection, with seroprevalence of 21.3% for HAV and 26.6% for HEV, highlighting the continued regional burden of these infections [11]. Rotavirus also remains an important cause of acute gastroenteritis in Rajasthan. Jain et al. and Meel et al. reported rotavirus positivity of 12.9% and 23.1%, respectively, among hospitalized children with acute diarrhea, with infections occurring throughout the year and showing a marked seasonal peak during the winter months [7,12]. Collectively, these findings indicate that HAV, HEV, and rotavirus account for a substantial proportion of laboratory-confirmed enterically transmitted viral infections encountered at tertiary care hospitals in Rajasthan, supporting their inclusion in regional laboratory-based epidemiological surveillance.

Although HAV, HEV, and rotavirus belong to different viral families and cause distinct clinical syndromes, they were included together in the present study based on epidemiological rather than virological considerations. They share a common fecal-oral mode of transmission, are closely associated with inadequate water, sanitation, and hygiene, require laboratory confirmation for diagnosis, and exhibit characteristic seasonal patterns with increased transmission during specific periods of the year. Furthermore, they are routinely investigated in our microbiology laboratory as part of the diagnostic workup of patients presenting with suspected acute viral hepatitis or acute gastroenteritis.

The burden of HAV, HEV, and rotavirus infections varies across geographical regions and is influenced by factors such as sanitation, hygiene, water quality, socioeconomic conditions, healthcare-seeking behavior, and access to laboratory testing. Although studies from different regions of India have described the epidemiology of acute viral hepatitis and rotavirus gastroenteritis, comprehensive data from Rajasthan evaluating these laboratory-confirmed enterically transmitted viral infections within a single tertiary care setting remain limited. Against this background, the present study was undertaken to describe the laboratory-confirmed burden, seasonal distribution, and demographic, socioenvironmental, and clinical characteristics of HAV, HEV, and rotavirus infections among eligible patients tested at our tertiary care center, rather than to compare their biological behavior.

## Materials and methods

Study design and setting

This prospective observational laboratory-based study was conducted at RVRS (Rajmata Vijaya Raje Scindia) Government Medical College, Bhilwara, Rajasthan, India, a tertiary care center serving Eastern Rajasthan. The study was conducted from January 2025 to March 2026. The Institutional Ethics Committee of RVRS Government Medical College, Bhilwara, Rajasthan, approved the original study protocol on November 10, 2021 (Ref No. RVRSMC/Ethics/2021/01). The study was conducted in accordance with the ethical principles of the Declaration of Helsinki.

Study population

A hospital-based consecutive sampling strategy was used. All eligible patients attending the Departments of Medicine and Pediatrics during the study period who fulfilled the inclusion criteria and underwent laboratory testing for clinically suspected acute viral hepatitis and/or acute diarrheal illness were enrolled consecutively. Acute diarrhea was defined as the passage of loose or watery stools for less than 14 days. This definition was based on the Indian Council of Medical Research (ICMR) guidelines for acute diarrheal disease [13]. Patients with clinically suspected acute viral hepatitis, including those with jaundice, dark urine, nausea/vomiting, loss of appetite, or other hepatic symptoms, underwent testing for HAV and/or HEV as clinically indicated. Patients with acute gastroenteritis, particularly those with watery diarrhea, vomiting, fever, or abdominal pain, underwent testing for rotavirus as clinically indicated. Patients with bloody diarrhea, dysentery, chronic diarrhea, clinically suspected bacterial or protozoal diarrhea, non-infectious causes of diarrhea, or incomplete essential laboratory information were excluded. All eligible patients tested for a specific virus were included in the denominator for virus-specific laboratory positivity. Only first-time laboratory-confirmed positive cases during the study period were included in the virus-specific demographic, socioenvironmental, clinical, and temporal analyses.

Laboratory testing

For suspected acute viral hepatitis, venous blood samples were collected and tested for anti-HAV immunoglobulin M (IgM) and anti-HEV IgM antibodies using enzyme-linked immunosorbent assay (ELISA) kits according to the manufacturers' instructions. Anti-HAV IgM and anti-HEV IgM antibodies were detected using the RecombiLISA™ IgM ELISA Kit (CTK Biotech, Inc., Poway, CA, USA). A positive anti-HAV IgM or anti-HEV IgM result was considered laboratory confirmation of recent HAV or HEV infection. For suspected acute viral gastroenteritis, fresh stool samples were collected in sterile containers and tested for rotavirus antigen using the OnSite® Rotavirus Ag Rapid Test CE (CTK Biotech, Inc., Poway, CA, USA) according to the manufacturer's instructions. A positive stool antigen result was considered laboratory confirmation of rotavirus infection. A total of 3,038 eligible patients were tested for anti-HEV IgM, 2,574 for anti-HAV IgM, and 560 for rotavirus stool antigen.

Data collection

Demographic, clinical, and exposure-related information was recorded prospectively using a structured case record form at the time of patient evaluation (Appendix 1). Variables included age, sex, residence, socioeconomic class, source of drinking water, history of open defecation, and clinical manifestations, including fever, nausea/vomiting, loss of appetite, jaundice, dark urine, and abdominal pain. Laboratory registers were used to verify test results and ensure that only first-time positive cases were included in the case analysis. Patients were analyzed according to virus-specific laboratory results. No laboratory-confirmed HAV-HEV co-infections were identified during the study period. Evaluation for bacterial enteric pathogens was beyond the scope of this study. For temporal analysis, laboratory-confirmed positive cases were grouped into five quarters: January-March 2025, April-June 2025, July-September 2025, October-December 2025, and January-March 2026.

Outcome measures

The primary outcome was virus-specific laboratory positivity, calculated as: $\text{Laboratory positivity (\%)}=\frac{\text{Number of newly laboratory-confirmed positive cases}}{\text{Number of eligible patients tested for that virus}}\times100$

Secondary outcomes were the quarterly distribution of laboratory-confirmed cases and the demographic, socioenvironmental, and clinical characteristics of HAV-, HEV-, and rotavirus-positive cases.

Statistical analysis

Data were entered into Microsoft Excel (Microsoft® Corp., Redmond, WA, USA) and analyzed using IBM SPSS Statistics for Windows, Version 27.0 (Released 2019; IBM Corp., Armonk, NY, USA). Categorical variables were presented as frequency and percentage. Virus-specific laboratory positivity was reported with 95% confidence intervals (CIs). Continuous variables were summarized using mean ± standard deviation (SD), median with interquartile range (IQR), and range. Age was compared across viral etiologies using the Kruskal-Wallis test because of non-normal distribution. Categorical variables were compared using Pearson’s chi-square test or Fisher’s exact test, as appropriate. A two-sided p-value of <0.05 was considered statistically significant. The reported 95% CIs describe the precision of the observed laboratory positivity within the study sample and should not be interpreted as estimates of population prevalence.

## Results

Among the eligible patients tested, rotavirus showed the highest laboratory positivity, with 80 of 560 patients testing positive (14.3%; 95% CI: 11.5-17.4), followed by HAV, with 350 of 2,574 patients testing positive (13.6%; 95% CI: 12.3-15.0). HEV positivity was observed in 319 of 3,038 patients (10.5%; 95% CI: 9.4-11.7) (Table 1).

**Table 1:** Virus-specific laboratory-confirmed positivity among eligible patients tested. ELISA: enzyme-linked immunosorbent assay

Viral etiology	Laboratory marker	New positive cases, n	Eligible patients tested, n	Positivity, % (95% CI)
Hepatitis E virus (HEV)	Anti-HEV IgM by ELISA	319	3,038	10.5 (9.4-11.7)
Hepatitis A virus (HAV)	Anti-HAV IgM by ELISA	350	2,574	13.6 (12.3-15.0)
Rotavirus	Stool antigen	80	560	14.3 (11.5-17.4)

Across the study period, the highest overall number of laboratory-confirmed cases was observed during Q4 (October-December 2025), with 227 of 749 cases (30.3%), followed by Q3 (July-September 2025), with 203 cases (27.1%). HEV cases were most frequent in Q3, accounting for 113 of 319 cases (35.4%), whereas HAV cases were highest in Q5 (January-March 2026), with 123 of 350 cases (35.1%). Rotavirus cases showed a marked peak in Q4, with 56 of 80 cases (70.0%) (Table 2).

**Table 2:** Quarterly distribution of laboratory-confirmed HEV, HAV, and rotavirus cases. HEV: hepatitis E virus; HAV: hepatitis A virus

Quarter	Months	HEV, n (%)	HAV, n (%)	Rotavirus, n (%)	Total, n (%)
Q1	Jan-Mar 2025	5 (1.6)	16 (4.6)	0 (0.0)	21 (2.8)
Q2	Apr-Jun 2025	51 (16.0)	46 (13.1)	6 (7.5)	103 (13.8)
Q3	Jul-Sep 2025	113 (35.4)	72 (20.6)	18 (22.5)	203 (27.1)
Q4	Oct-Dec 2025	78 (24.5)	93 (26.6)	56 (70.0)	227 (30.3)
Q5	Jan-Mar 2026	72 (22.6)	123 (35.1)	0 (0.0)	195 (26.0)
Total	Jan 2025-Mar 2026	319 (100.0)	350 (100.0)	80 (100.0)	749 (100.0)

The mean age was highest among HEV cases (19.45 ± 10.31 years), followed by HAV cases (17.96 ± 13.49 years), while rotavirus cases were markedly younger (4.21 ± 1.59 years). The median age was 19 years (IQR: 11-27) for HEV, 14 years (IQR: 8-24) for HAV, and four years (IQR: 3-5) for rotavirus cases. Age differed significantly across the three viral etiologies (Kruskal-Wallis H = 99.64, p < 0.001). Male patients predominated among HAV cases (222/350, 63.4%) and rotavirus cases (50/80, 62.5%), whereas females predominated among HEV cases (199/319, 62.4%). The sex distribution differed significantly across the viral etiologies (χ² = 48.28, p < 0.001). Most HAV cases (240/350, 68.6%) and HEV cases (171/319, 53.6%) were aged 0-20 years, while all rotavirus cases were in this age group (80/80, 100.0%) (Table 3).

**Table 3:** Demographic characteristics of laboratory-confirmed cases by viral etiology. HEV: hepatitis E virus; HAV: hepatitis A virus

Characteristic	Domain	HAV (n = 350)	HEV (n = 319)	Rotavirus (n = 80)	Test statistic	p-value
Age, years, mean ± SD		17.96 ± 13.49	19.45 ± 10.31	4.21 ± 1.59	Kruskal-Wallis H = 99.64	<0.001
Age, years, median (IQR)		14 (8-24)	19 (11-27)	4 (3-5)	-	-
Age range, years		3-65	3-55	2-8	-	-
Sex	Female	128 (36.6)	199 (62.4)	30 (37.5)	χ²(2) = 48.28	<0.001
	Male	222 (63.4)	120 (37.6)	50 (62.5)		
Age group	0-20 years	240 (68.6)	171 (53.6)	80 (100.0)	χ²(4) = 84.27	<0.001
	21-40 years	85 (24.3)	141 (44.2)	0 (0.0)		
	≥41 years	25 (7.1)	7 (2.2)	0 (0.0)		

Slum residence was the most common residential category among HAV cases (172/350, 49.1%) and HEV cases (131/319, 41.1%), whereas rural residence was most common among rotavirus cases (35/80, 43.8%). Residence distribution did not differ significantly across the three viral etiologies (χ² = 6.51, p = 0.164). Lower-middle and middle socioeconomic classes accounted for the largest proportion of cases across all viral etiologies. Socioeconomic class distribution was comparable among HAV, HEV, and rotavirus cases (χ² = 4.39, p = 0.624). Drinking-water source also did not differ significantly across the viral etiologies (χ² = 14.33, p = 0.074), although bore-well water was more frequently reported among rotavirus cases (26/80, 32.5%). Open defecation was reported by 107/350 (30.6%) HAV cases and 96/319 (30.1%) HEV cases, compared with 10/80 (12.5%) rotavirus cases. This distribution differed significantly across the three viral etiologies (χ² = 11.20, p = 0.004) (Table 4).

**Table 4:** Socioenvironmental characteristics of laboratory-confirmed cases by viral etiology. HEV: hepatitis E virus; HAV: hepatitis A virus

Variable	Category	HAV (n = 350)	HEV (n = 319)	Rotavirus (n = 80)	Test statistic	p-value
Residence	Rural	117 (33.4)	126 (39.5)	35 (43.8)	χ²(4) = 6.51	0.164
	Slum	172 (49.1)	131 (41.1)	30 (37.5)		
	Urban	61 (17.4)	62 (19.4)	15 (18.8)		
Socioeconomic class	Lower	61 (17.4)	47 (14.7)	9 (11.3)	χ²(6) = 4.39	0.624
	Lower middle	110 (31.4)	107 (33.5)	22 (27.5)		
	Middle	110 (31.4)	96 (30.1)	30 (37.5)		
	Upper middle	69 (19.7)	69 (21.6)	19 (23.8)		
Drinking-water source	Bore-well	56 (16.0)	62 (19.4)	26 (32.5)	χ²(8) = 14.33	0.074
	Filtered	62 (17.7)	54 (16.9)	15 (18.8)		
	Handpump	72 (20.6)	63 (19.7)	15 (18.8)		
	Piped	81 (23.1)	69 (21.6)	9 (11.3)		
	Tank	79 (22.6)	71 (22.3)	15 (18.8)		
Open defecation	No	243 (69.4)	223 (69.9)	70 (87.5)	χ²(2) = 11.20	0.004
	Yes	107 (30.6)	96 (30.1)	10 (12.5)		

Fever was reported in a similar proportion of HAV (207/350, 59.1%), HEV (201/319, 63.0%), and rotavirus cases (49/80, 61.3%), with no significant difference across the groups (χ² = 1.05, p = 0.591). Nausea/vomiting was more frequent among HAV (305/350, 87.1%) and HEV cases (265/319, 83.1%) than rotavirus cases (43/80, 53.8%) (χ² = 71.30, p < 0.001). Loss of appetite was reported in 292/350 (83.4%) HAV cases and 254/319 (79.6%) HEV cases, while none of the rotavirus cases had loss of appetite (χ² = 286.31, p < 0.001). Jaundice was present in 177/350 (50.6%) HAV cases and 170/319 (53.3%) HEV cases, whereas no rotavirus case had jaundice (χ² = 127.11, p < 0.001). Dark urine was reported in 128/350 (36.6%) HAV cases and 99/319 (31.0%) HEV cases, with no cases among the rotavirus group (χ² = 62.34, p < 0.001). Abdominal pain was most frequent among rotavirus cases (49/80, 61.3%), compared with HAV (99/350, 28.3%) and HEV cases (60/319, 18.8%) (χ² = 57.52, p < 0.001) (Table 5).

**Table 5:** Clinical manifestations according to viral etiology. HEV: hepatitis E virus; HAV: hepatitis A virus

Clinical manifestation	HAV (n = 350)	HEV (n = 319)	Rotavirus (n = 80)	Test statistic	p-value
Fever	207 (59.1)	201 (63.0)	49 (61.3)	χ²(2) = 1.05	0.591
Nausea/vomiting	305 (87.1)	265 (83.1)	43 (53.8)	χ²(2) = 71.30	<0.001
Loss of appetite	292 (83.4)	254 (79.6)	0 (0.0)	χ²(2) = 286.31	<0.001
Jaundice	177 (50.6)	170 (53.3)	0 (0.0)	χ²(2) = 127.11	<0.001
Dark urine	128 (36.6)	99 (31.0)	0 (0.0)	χ²(2) = 62.34	<0.001
Abdominal pain	99 (28.3)	60 (18.8)	49 (61.3)	χ²(2) = 57.52	<0.001

## Discussion

This study evaluated the laboratory-confirmed burden and clinico-epidemiological profile of HAV, HEV, and rotavirus infections among eligible patients tested at a tertiary care hospital in Eastern Rajasthan. HAV, HEV, and rotavirus are important fecal-oral viral pathogens, but their distribution may vary according to age, sex, season, sanitation-related exposures, and local healthcare-seeking patterns. The present findings provide context-specific information on positivity rates, seasonal trends, demographic characteristics, environmental factors, and clinical manifestations of these infections.

In this study, rotavirus had the highest laboratory positivity, detected in 80 of 560 eligible patients (14.3%; 95% CI: 11.5-17.4), followed by HAV in 350 of 2,574 patients (13.6%; 95% CI: 12.3-15.0) and HEV in 319 of 3,038 patients (10.5%; 95% CI: 9.4-11.7). The HAV and HEV positivity rates were similar to those reported by Soni et al. from Rajasthan, who observed HAV and HEV positivity of 12.7% and 10.9%, respectively [2]. Jain et al. reported a comparable rotavirus positivity of 12.9% among hospitalized children in South Rajasthan [7]. However, higher rotavirus positivity was reported by Seema et al. in Eastern India (27.1%) and Meel et al. in Western Rajasthan (23.1%) [12,14]. Variations in positivity rates across studies may reflect differences in age groups, clinical setting, enrollment criteria, study duration, and diagnostic testing practices.

The overall number of laboratory-confirmed cases was highest during October-December 2025 (227/749, 30.3%), followed by July-September 2025 (203/749, 27.1%). HEV cases were most frequent during July-September 2025 (113/319, 35.4%), whereas HAV cases were highest during January-March 2026 (123/350, 35.1%). Rotavirus showed a marked peak during October-December 2025, accounting for 56 of 80 cases (70.0%). The monsoon-associated increase in HEV cases was similar to observations by Soni et al., Palewar et al., Sharma et al., and Netra et al., who reported increased HAV and/or HEV cases during monsoon or post-monsoon months [2,15-17]. The winter peak of rotavirus in the present study was consistent with findings from Jain et al. and Meel et al., who reported higher rotavirus occurrence during December-February and November-February, respectively [7,12]. The later peak of HAV in the present study may indicate local variation in exposure patterns and transmission dynamics.

Age differed significantly across the three viral etiologies (Kruskal-Wallis H = 99.64, p < 0.001). HEV cases had the highest mean age (19.45 ± 10.31 years), followed by HAV cases (17.96 ± 13.49 years), while rotavirus cases were substantially younger (4.21 ± 1.59 years). Most HAV cases were aged 0-20 years (240/350, 68.6%), supporting earlier studies by Soni et al., Palewar et al., Sharma et al., and Netra et al., which also found HAV predominantly among children and adolescents [2,15-17]. In contrast, although 53.6% of HEV cases in the present study were aged 0-20 years, several studies have reported HEV more commonly among young adults, particularly those aged 21-40 years [2,15-17]. This difference may be related to the population tested, local exposure patterns, healthcare-seeking behavior, referral patterns at our tertiary care center, or changing epidemiology of enterically transmitted hepatitis. All rotavirus cases in the present study were aged 0-20 years, with a median age of four years. However, previous studies from Rajasthan and other Indian settings have commonly reported the greatest rotavirus burden among children below two years of age [7,12,14]. This difference should be interpreted cautiously because the present study included children up to eight years of age and did not restrict testing to children under five years.

Male predominance was observed among HAV cases (222/350, 63.4%) and rotavirus cases (50/80, 62.5%), whereas females predominated among HEV cases (199/319, 62.4%); the difference in sex distribution across viral etiologies was statistically significant (χ² = 48.28, p < 0.001). The male predominance in HAV cases was consistent with findings from Soni et al., Sharma et al., and Netra et al. [2,15,17]. Similarly, male predominance among rotavirus cases was consistent with reports by Jain et al., Seema et al., and Meel et al. [7,12,14]. Female predominance among HEV cases was similar to the findings of Soni et al. and Palewar et al. [2,16], although other studies have reported male predominance in HEV cases [15,17]. These differences may be influenced by demographic composition, health-care-seeking behavior, and local patterns of exposure.

Slum residence was the most common residential category among HAV cases (172/350, 49.1%) and HEV cases (131/319, 41.1%), whereas rural residence was most common among rotavirus cases (35/80, 43.8%). However, residence distribution did not differ significantly across the three viral etiologies (χ² = 6.51, p = 0.164). Soni et al. also reported a greater number of HAV and HEV cases among people living in slum areas, supporting the pattern observed in the present study [2]. Lower-middle and middle socioeconomic classes accounted for the largest proportion of cases across all viral etiologies, and socioeconomic class did not differ significantly between groups (χ² = 4.39, p = 0.624). This differed from Soni et al., who reported a higher proportion of HAV and HEV cases in the low socioeconomic group [2]. Differences in socioeconomic classification, study populations, and local living conditions may explain this variation.

Drinking-water source did not differ significantly across the three viral etiologies (χ² = 14.33, p = 0.074). Bore-well water was reported by 16.0% of HAV cases, 19.4% of HEV cases, and 32.5% of rotavirus cases. Similarly, Soni et al. and Meel et al. found no significant association between drinking-water source and viral infection [2,12]. Open defecation was reported more frequently among HAV and HEV cases than among rotavirus cases (χ² = 11.20, p = 0.004). This difference may reflect variation in sanitation-related exposures across the three laboratory-confirmed groups; however, no causal association can be inferred from this study.

Fever was reported in similar proportions of HAV (59.1%), HEV (63.0%), and rotavirus cases (61.3%), with no significant difference across groups (χ² = 1.05, p = 0.591). Nausea/vomiting was more frequent in HAV (87.1%) and HEV (83.1%) than in rotavirus cases (53.8%) (p < 0.001). Loss of appetite, jaundice, and dark urine were observed among HAV and HEV cases but were absent among rotavirus cases (all p < 0.001), consistent with the hepatotropic nature of HAV and HEV infection. Abdominal pain was most frequent among rotavirus cases (61.3%), compared with HAV (28.3%) and HEV (18.8%) cases (p < 0.001). Previous rotavirus studies also reported vomiting and fever as common manifestations, although their frequency varied across settings [7,12,14]. The higher frequency of abdominal pain in the present rotavirus group compared with some earlier reports may reflect differences in symptom reporting, age distribution, or clinical severity at presentation.

Overall, the study demonstrates a substantial burden of laboratory-confirmed HAV, HEV, and rotavirus infections among eligible patients in Eastern Rajasthan. The distinct seasonal, demographic, and clinical patterns observed across the three etiologies may help guide targeted testing, preventive counseling regarding hygiene and sanitation, and timely clinical management. As a limitation, this single-center, hospital-based study may not represent the wider community. Positivity rates reflect only eligible patients who were tested and should not be interpreted as population prevalence. Differences in the number and characteristics of patients tested across viral etiologies limit direct comparisons. The study did not assess other enteric pathogens, viral genotypes, vaccination status, disease severity, or clinical outcomes. The study did not evaluate bacterial enteric pathogens or mixed viral-bacterial co-infections. Quarter-wise testing denominators were not available; therefore, quarterly positivity rates could not be calculated.

## Conclusions

This study demonstrates that HAV, HEV, and rotavirus continue to contribute substantially to the burden of enterically transmitted viral infections in Eastern Rajasthan, consistent with the regional epidemiological pattern reported from other parts of the state. Rotavirus showed the highest laboratory positivity, followed by HAV and HEV, while each infection exhibited distinct seasonal and demographic patterns. These findings reinforce the importance of continued laboratory-based surveillance for major enterically transmitted viral infections to monitor disease trends and guide public health interventions. Strengthening water, sanitation, and hygiene measures, along with timely laboratory diagnosis and appropriate preventive strategies, remains essential to reducing the burden of these infections in the region.

## Appendices

Appendix 1

**Table 6:** A structured case record form.

Section	Variables
Patient ID	
Age	
Sex	Male/Female
Residence	Rural/Slum/Urban
Socioeconomic status	Lower/Lower middle/Middle/Upper middle
Drinking water source	Bore-well//Filtered/Handpump/Piped/Tank
Open defecation	Yes/No
Fever	Yes/No
Nausea/Vomiting	Yes/No
Loss of appetite	Yes/No
Jaundice	Yes/No
Dark urine	Yes/No
Abdominal pain	Yes/No
Anti-HAV IgM	Positive/Negative
Anti-HEV IgM	Positive/Negative
Rotavirus antigen	Positive/Negative
